# The use of telepathology in veterinary medicine: a scoping review

**DOI:** 10.1177/10406387241241270

**Published:** 2024-05-14

**Authors:** Lindsay Rogers, Angelica Galezowski, Heather Ganshorn, Dayna Goldsmith, Carolyn Legge, Katie Waine, Erin Zachar, Jennifer L. Davies

**Affiliations:** Faculty of Veterinary Medicine, University of Calgary, Calgary, Alberta, Canada; Faculty of Veterinary Medicine, University of Calgary, Calgary, Alberta, Canada; Library and Cultural Resources, University of Calgary, Calgary, Alberta, Canada; Faculty of Veterinary Medicine, University of Calgary, Calgary, Alberta, Canada; Faculty of Veterinary Medicine, University of Calgary, Calgary, Alberta, Canada; Faculty of Veterinary Medicine, University of Calgary, Calgary, Alberta, Canada; Faculty of Veterinary Medicine, University of Calgary, Calgary, Alberta, Canada; Faculty of Veterinary Medicine, University of Calgary, Calgary, Alberta, Canada

**Keywords:** laboratories, microscopy, pathologists, telepathology, telecommunications, veterinarians

## Abstract

Telepathology, as a subset of teleconsulting, is pathology interpretation performed at a distance. Telepathology is not a new phenomenon, but since ~2015, significant advances in information technology and telecommunications coupled with the pandemic have led to unprecedented sophistication, accessibility, and use of telepathology in human and veterinary medicine. Furthermore, telepathology can connect veterinary practices to distant laboratories and provide support for underserved animals and communities. Through our scoping review, we provide an overview of how telepathology is being used in veterinary medicine, identify gaps in the literature, and highlight future areas of research and service development. We searched MEDLINE, CAB Abstracts, and the gray literature, and included all relevant literature. Despite the widespread use of digital microscopy in large veterinary diagnostic laboratories, we identified a paucity of literature describing the use of telepathology in veterinary medicine, with a significant gap in studies addressing the validation of whole-slide imaging for primary diagnosis. Underutilization of telepathology to support postmortem examinations conducted in the field was also identified, which indicates a potential area for service development. The use of telepathology is increasing in veterinary medicine, and pathologists must keep pace with the changing technology, ensure the validation of innovative technologies, and identify novel uses to advance the profession.

Teleconsulting is not a new phenomenon, and it is increasingly used in veterinary medicine as technology improves. Furthermore, teleconsulting gained prominence during the COVID-19 pandemic as veterinary clinics looked for alternatives to in-person consultation.^7^ Telepathology is a subset of teleconsulting, defined as performing pathology interpretation at a distance.^12,33^ Although the term *telepathology* was first introduced in 1986, the earliest recorded instance of telepathology occurred in 1969 when microwave-based telecommunication was used to transmit live black-and-white images of histology slides and blood smears.^3,32^ Fifty years later, there have been significant advances in information technology and telecommunications; as a result, telepathology has become more sophisticated, more readily accessible, and more frequently used in human and veterinary medicine.^6,21^

Telepathology involves the acquisition of cytologic, hematologic, histologic, or macroscopic images for transmission along telecommunication pathways for diagnosis, consultation, education, and research.^3^ This process can include *static* telepathology, also known as offline or store-and-forward, and *dynamic* or “real-time” pathology.^8,20,31^ Digital microscopy (DM) can be further separated into robotic microscopy, region of interest (ROI) digital microscopy, or whole-slide imaging (WSI). Visualization of the sample on a computer is the common thread that links these modalities, but they differ in how much of the slide is available for viewing (individual fields, ROIs, or the entire slide) and whether the image is stored (static telepathology, WSI, or ROI scans) or viewed in “real-time” (robotic microscopy).^8,31^

When first described in the human pathology literature, the role of telepathology in distributing much-needed pathology expertise to rural areas was emphasized.^32^ In North America, many rural veterinary practices are geographically distant from the laboratory and veterinary specialists, making teleconsulting particularly valuable. These clinics serve all species of animals, and rapid test results are key to client satisfaction and timely therapeutic decisions.^24^ Additionally, rapid results are vital in production animal operations in which understanding the cause of disease is paramount not only to the overall health of the herd but also to identification of emerging, zoonotic, or foreign animal diseases; food safety; and public health.^1^ Poor access to testing can result in delayed or empirical treatment, inappropriate use of antimicrobials, increased risk of disease outbreaks, and diminished passive surveillance. Further understanding of current telepathology technology and use may provide increased accessibility to diagnostic services for these communities and help direct future research.

Our objective was to complete a scoping review to summarize findings on telepathology in veterinary medicine and to identify gaps in the research literature and telepathology services to aid in the planning of future research projects.

## Methods

### Protocols and registration

We prepared the protocol for this scoping review using the Preferred Reporting Items for Systematic Reviews and Meta-Analysis for Scoping Reviews (PRISMA-ScR) reporting guidelines (Suppl. Material) and published the protocol on the University of Calgary PRISM repository^25^ and at SYREAF (www.syreaf.org).^30^

### Eligibility criteria

To be eligible for inclusion, articles and gray literature had to be:

1. Available in English in full text but could be any type of research article or relevant gray literature including blogs, websites, and information pamphlets.
2. Published after the year 2000.
3. Investigating veterinary telepathology, defined as “performing veterinary pathology interpretation at a distance,” which included digital or virtual gross pathology, histology, cytology, and hematology.^12,33^ This could be by digital still images, digital microscopy, or video consulting.

### Information sources

We searched the following databases for relevant studies: CAB Abstracts and MEDLINE. We loaded the articles onto Covidence (https://www.covidence.org/) for de-duplication and screening.

We searched the following websites for relevant gray literature: national veterinary medical associations that are members of the World Veterinary Association (e.g., Canadian Veterinary Medical Association, CVMA), veterinary pathologist (anatomic and clinical) membership websites (e.g., American Association of Veterinary Laboratory Diagnosticians, AAVLD), international animal health organizations (e.g., World Organisation for Animal Health, WOAH), and global commercial veterinary diagnostic laboratories (e.g., Idexx Laboratories). We also searched through references from included articles for additional relevant literature. Following consultation with a librarian, we opted to not use general search engines, such as Google, due to the lack of reproducible searches from these platforms.

### Search

The search strategies for MEDLINE and CAB Abstracts were drafted by a librarian with input from content experts (Tables 1, 2). We performed the initial database search on 2021 Oct 15, with additional searches completed 2022 Jun 11, 2023 Aug 2, and 2023 Sep 29.

**Table 1.** MEDLINE database search strategy used for our scoping review on the use of telepathology in veterinary medicine.

	Searches	Results	Type
1	exp Remote Consultation/	5,444	Advanced
2	exp Telemedicine/	37,532	Advanced
3	("remote consult*" or "virtual consult*" or telemedicine).kf,tw.	18,982	Advanced
4	1 or 2 or 3	44,797	Advanced
5	exp Microscopy/	564,882	Advanced
6	exp Image Processing, Computer-Assisted/	247,450	Advanced
7	(patholog* or microscop* or cytolog* or cytopatholog* or "digital h?ematolog*" or imaging or scanning or (imag* adj2 analy*) or necrops*).kf,tw.	2,628,091	Advanced
8	5 or 6 or 7	3,006,517	Advanced
9	4 and 8	3,636	Advanced
10	exp Telepathology/	894	Advanced
11	(telepatholog* or telecyto* or "remote patholog*" or "remote cyto*").kf,tw.	873	Advanced
12	10 or 11	1,254	Advanced
13	9 or 12	4,103	Advanced
14	exp Veterinary Medicine/	25,612	Advanced
15	exp Livestock/	4,459	Advanced
16	exp Cattle Diseases/ or exp Cattle/	360,155	Advanced
17	exp Horses/ or exp Horse Diseases/	72,548	Advanced
18	exp Sheep Diseases/ or exp Sheep/	124,318	Advanced
19	exp Swine/ or exp Swine Diseases/	231,100	Advanced
20	exp Poultry Diseases/ or exp Poultry/	159,294	Advanced
21	exp Dogs/ or exp Cats/	448,368	Advanced
22	(veterinary or livestock or cattle or horse* or sheep or swine or pig or pigs or poultry or chicken* or turkey* or dog? or cat? or canine* or feline*).kf,tw.	1,097,815	Advanced
23	14 or 15 or 16 or 17 or 18 or 19 or 20 or 21 or 22	1,720,449	Advanced
24	13 and 23	39	Advanced

**Table 2.** CAB Abstracts (Ebsco) database search strategy used for our scoping review on the use of telepathology in veterinary medicine.

	Query	Results
1	("remote consult*" or "virtual consult*" or telemedicine) AND (patholog* or microscop* or cytolog* or cytopatholog* or "digital h#ematolog*" or imaging or scanning or (imag* N2 analy*) or necrops*)	110
2	telepatholog* or telecyto* or "remote patholog*" or "remote cyto*"	32
3	S1 OR S2	133
4	veterinary or livestock or cattle or horse* or sheep or swine or pig or pigs or poultry or chicken* or turkey*	2,196,795
5	S3 AND S4	25

### Source selection

Two reviewers (L. Rogers, J.L. Davies) selected relevant studies and gray literature using Covidence to manage the literature. Each reviewer independently reviewed the literature. The reviewers discussed conflicts after all articles had been reviewed. If no consensus was reached, a discussion with the team of content experts was initiated for consensus. We tested the review process with the first 10% of articles and no amendment was needed. The same process was applied to both published articles and gray literature, except where no abstract was available. In these cases, we applied the criteria for the abstract to the entire article. There were 3 stages to literature selection:

1. Title review: we left this step intentionally broad to capture as much relevant literature as possible. We excluded titles containing keywords indicating that the article was explicitly about topics other than veterinary telepathology. We used the keyword indicator in Covidence to assist with selection. Keywords for article exclusion were:
  a. Bacteriology
  b. Human medicine
  c. Radiology or teleradiology
2. Abstract review: we reviewed the abstract, and selected literature according to the following questions.
  a. Is the literature relevant to veterinary medicine?
    i. Yes or maybe: continue to question b.
    ii. No: exclude
  b. Is the literature about radiology, bacteriology, or in the context of human medicine?
    i. Yes: exclude
    ii. No or maybe: continue to question c.
  c. Is telepathology, the performance of pathology interpretation at a distance, performed or discussed in the literature?
    i. Yes or maybe: include
    ii. No: exclude
3. Full-text review: we completed full-text review of literature simultaneously with data extraction. Any articles that did not meet inclusion criteria applied to the abstract in step 2 were excluded after discussion between the 2 independent reviewers.

Articles in which authors investigated the use of digital histopathology, cytology, hematology, or postmortem examination solely for teaching students were not considered the performance of pathology interpretation at a distance and thus were excluded according to step 2c.

### Data charting process

The same 2 independent reviewers charted data in Excel (Microsoft) and tested the data charting process with the first 10% of articles to ensure clarity and consistency. No revisions were necessary after the testing. Any conflicts were resolved between the 2 reviewers. If consensus was not reached, the conflict was brought to the team of content experts for review. A single reviewer compiled the charted data for synthesis.

### Data items

We extracted the following data items from the literature.

- Country of corresponding author
- Study design
- Purpose of study or publication
- Division of pathology: gross pathology, histopathology, cytology/hematology
- End-users: pathologists, education or students, consulting, veterinarians, producers, or owners
- Whether the telepathology was performed in static, real-time, or by ROI, or WSI, as defined below:
  ○ Static telepathology: storage and transfer of static images (i.e., photographs of microscope slides or postmortems) for analysis at a time other than the actual viewing of the slide or postmortem.^8,9^
  ○ Real-time telepathology: any telepathology in which the postmortem or microscope slide is being viewed concurrently by the referring veterinarian and anatomic or clinical pathologist, including robotic microscopy.^8,31^
  ○ ROI digital microscopy: a subset of digital microscopy that uses image-stitching software to create a low magnification image of a slide, then selects ROI at higher magnification, and stitches the images into a small file of embedded magnifications.^8^
  ○ WSI: digitization of an entire glass slide that uses software to create a digital image. Software emulates the use of a light microscope on the computer.^9^

- Form of communication used: telephone, email, video conferencing, still-images, digital microscopy slides, or other
- Technology used for interpretation: digital or virtual still-images, digital or virtual microscopy slides, real-time video, post-procedural video, written or oral description, or other
- Summary of overall conclusions

### Critical appraisal

We did not perform a critical appraisal of the literature because this was a scoping review.

### Synthesis of results

We used descriptive statistics to summarize the findings, presented via a combination of tables, figures, and descriptive text. We identified and discussed literature gaps and potential novel means of telepathology.

## Results

**Figure 1.**
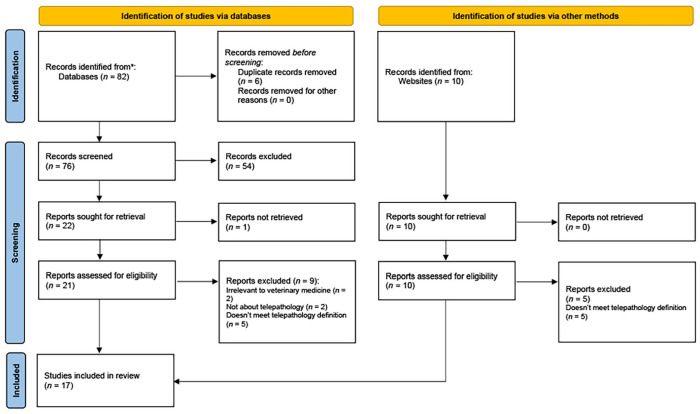
Number (*n*) of articles identified, screened, and included for our scoping review on the use of telepathology in veterinary medicine. From: Page MJ, et al. The PRISMA 2020 statement: an updated guideline for reporting systematic reviews. BMJ 2021;372:n71. doi: 10.1136/bmj.n71. For more information, visit: http://www.prisma-statement.org/

Following de-duplication, we screened 86 pieces of literature and included 17 articles^1–3,5,6,8–11,15,17,19,20,24,27,29,31^ after the full-text review (Fig. 1). Of these, 8 were primary research articles, 2 were conference proceedings, 2 were review articles, and 5 were gray literature. All articles were published in 2003 or later. Corresponding authors were from 7 different countries: Canada (2), Germany (2), India (1), Italy (3), Sweden (1), United Kingdom (3), and USA (5; Table 3).

**Table 3.** Summary of characteristics and study data for included literature for our scoping review on the use of telepathology in veterinary medicine.

Article	Country of corresponding author							Study design						Division of pathology			End-users			Mode				Communication				Technology			Study purpose			Study conclusions			
	Canada	Germany	India	Italy	Sweden	UK	USA	Conference proceedings	Cross-sectional	Case report	Review	Case-control	Grey literature	Anatomic–Gross	Anatomic–Histology	Cytology/Hematology	Veterinarians	Students	Pathologists	Static	Real-time	Region of Interest	Whole slide images	App/online platform	Remote video	Email	Portal/shared drive	Digital images	Augmented reality	Digital microscopy	Evaluate & validate new technology	Describe novel use of telepathology	Review, overview, or inform	Telepathology has potential but requires refinement	Technology is effective where applied	There are major advantages and increasing use	Conventional microscopy is superior to telepathology
1							X	X						X			X			X				X				X			X			X			
2					X				X					X			X				X				X				X		X			X			
4			X							X					X		X	X	X	X						X		X				X			X		
6		X										X			X				X				X	X						X	X			X			
7		X									X				X	X		X	X				X	X						X			X			X	
9							X					X				X	X		X			X					X			X	X			X			
10				X								X				X			X				X	X						X	X			X			
11				X								X				X			X				X	X						X	X				X		
12							X					X				X	X		X	X							X	X			X						X
16						X							X		X	X			X				X	X						X			X			X	
18						X							X			X	X													X			X			X	
20	X												X			X	X			X						X		X					X			X	
21				X								X				X	X		X	X						X		X			X				X		
25						X							X		X	X			X				X	X						X			X			X	
26							X						X			X	X						X	X						X			X			X	
28	X							X								X	X			X						X		X					X		X		
30							X				X				X				X				X	X						X			X		X		
Total	2	2	1	3	1	3	5	2	1	1	2	6	5	2	6	12	10	2	11	6	1	1	8	9	1	4	2	6	1	10	8	1	8	5	5	6	1

We divided the study objectives for included articles into 3 broad categories: 1) to evaluate and validate a tool, application, or technique (8 articles); 2) to describe the novel use of telepathology (1 article); or 3) to review, overview, or inform potential users about the technology available for telepathology (8 articles; Table 3).

We found that the included literature covered 3 divisions of pathology, with some articles covering more than 1 division: gross pathology (2 articles), histopathology (6 articles), and cytology/hematology (12 articles). We found 4 modes of telepathology: static, real-time, ROI, or WSI. The mode in 6 articles was static telepathology; in 1 article, real-time telepathology; in 1 article, ROI; and in 8 articles WSI. The authors did not state the mode for 1 article. We found technology used for telepathology included digital photos (5 articles), augmented reality (1 article), and digital microscopy (10 articles). We found communication for telepathology was through an application or online platform (9 articles), remote video (1 article), email (4 articles), or a portal or shared drive (2 articles). The authors did not state the communication used in 1 article. The end-users of the telepathology techniques or technology were veterinarians only (6 articles), pathologists only (6 articles), or a combination of students, veterinarians, and/or pathologists (5 articles; Table 3).

We grouped findings from the included articles into 4 broad categories: 1) the technology or technique investigated has potential but requires refinement and/or development (5 articles); 2) the technology was effective and viable in the situations applied (5 articles); 3) the technology has major advantages and is increasingly being used (6 articles); or 4) the technology should not replace conventional microscopy (1 article; Table 3).

## Discussion

Interestingly, very few articles met our inclusion criteria, indicating a relative paucity of literature describing the use of telepathology in veterinary medicine compared to the increasing use of this technology within veterinary diagnostic laboratories.^15^ Most of the veterinary literature focused on microscopy, both cytology and histopathology, which is not surprising as development of digital microscopy techniques started in the mid-1980s for human medicine.^32^ Forms of DM used in veterinary medicine included static telepathology, ROI microscopy, and increasingly WSI. DM has been transformed by the development of WSI systems, and WSI is now considered superior to both static and robotic telepathology.^6,23^ DM has been adopted by veterinary pathologists for consulting, research, teaching, archiving, and increasingly primary diagnostic work in large laboratories using WSI as their primary technology for evaluation of histologic and cytologic slides.^6^

In medicine, systematic studies addressing the reliability of DM in daily routine pathology laboratories show that interactive DM has a high concordance comparable to light microscopy, and WSI technology has been validated for histopathology, cytology, and hematology.^4,13,16,18,22,28^ Furthermore, a commercial WSI histopathology system received U.S. Food and Drug Administration approval for primary diagnostic use in 2017. We found only 3 studies addressing the validation of WSI in veterinary pathology, with good agreement between WSI and light microscopy for the histologic diagnosis of canine cutaneous tumors,^5^ the evaluation of cytologic samples of canine lymphoma,^9^ and the evaluation of canine and feline cytologic samples.^10^ There is a paucity of WSI validation studies addressing the wide array of histopathology, cytology, and hematology samples seen at veterinary diagnostic laboratories, highlighting the need for future research as DM becomes more commonplace.

We identified more articles examining the use of DM for cytology than for histopathology and found similarities and differences in how the technology is used between the 2 disciplines. Common to both disciplines is the increasing usage of DM, specifically WSI, at diagnostic laboratories, allowing for rapid, worldwide transmission of pathology data for primary diagnosis and second opinions; global access to a pool of remote-working pathologists; digital archive of images for secure, potentially permanent, and rapid retrieval; resident training; image analysis; and the promise of artificial intelligence.^5,10,31^ However, with the ease of processing and staining of cytology and hematology slides in-clinic combined with more reliable and affordable equipment and telecommunication platforms, a unique feature of telecytology is its ability to connect the community practitioner to the clinical pathologist.^11,19,27,29^ Telecytology in community practice ranges from the use of static images to the use of in-clinic digital slide scanners to send images or scanned slides to a remote clinical pathologist.^11,19,27–29^ Thereby, telecytology accelerates the diagnostic process by eliminating the complexities and delays associated with shipping glass slides to the laboratory, resulting in rapid analysis and results. It is particularly suited to facilitating intra-operative diagnosis and decision-making.^29^ In contrast, the complexities of tissue processing and slide staining for histopathology samples require that specimens are still sent to the laboratory, rather than processed in-clinic, where slides can then be scanned and interpreted by an anatomic pathologist.^14,31^ Alongside the benefits listed above, DM use within the histotechnology laboratory has brought new challenges, including increased steps to convert the glass slide to a digital image with additional requirements for IT hardware and software, ongoing maintenance, and increased staff time.^6^ However, telepathology does allow increased connection between clinician and histopathologist where required, with cases more easily shared as captured images or by live sharing of computer screens.^14,31^

Only 2 of the articles that we included in our scoping review focused on gross anatomic telepathology, which reflects a lack of publication on its use in veterinary medicine. Those authors looked at the use of virtual feedlot postmortems through still-images and the use of virtual reality for meat inspection in pigs at the slaughterhouse.^1,2^ While specific in their use, these 2 studies are precursors to other potential uses of telepathology for postmortem examinations, particularly the use of augmented reality. In many regions of North America, large geographic distances between farms and diagnostic laboratories prohibit the submission of whole large animal carcasses for postmortem examination. As a result of this barrier and others, it is estimated that < 1% of cattle that die before slaughter undergo postmortem examination, representing a huge gap in surveillance.^1^ While still-images for gross anatomic telepathology or digital postmortem examinations certainly have utility, they are associated with significant limitations, including the static nature of the images and lag time between the postmortem and the examination of the digital photographs. These limitations leave clinicians and pathologists at a disadvantage. Not all organ systems may be examined, photographs may be biased by the prosector’s interpretation, and appropriate samples may not have been harvested for histopathology and ancillary testing. Video streaming and augmented reality can improve access to testing for these remote and/or underserved rural communities. Veterinarians serving these areas with minimal infrastructure or expertise in anatomic pathology could remotely connect with a pathologist to consult on difficult cases. Through real-time telepathology, the pathologist could assist the on-site veterinarian with the postmortem examination and consult on tissue sampling for further testing. Technicians and producers could also be trained in areas without consistent veterinary care to perform assisted field postmortem examinations. Indeed, both studies included in our review were similar in that they promoted the use of technicians for the on-site fieldwork, freeing the veterinarian or specialist to perform the diagnostic tasks.^1,2^ In the current period of food-animal and rural mixed-animal veterinarian shortages across North America, delegating more fieldwork to technical staff could be beneficial.^26^ It may increase the job satisfaction of technical staff by allowing them to perform higher-level tasks for which they can be trained, and can decrease the pressure on the veterinarian’s time while still meeting the producer’s needs. Improving access to testing is of tremendous benefit to the health and welfare of animals, public health, and food safety, especially in remote and underserved areas. The latter may be of particular importance for remote Indigenous communities that rely on subsistence hunting.

Unfortunately, the uptake of real-time gross telepathology is not without challenges, which may account for the gap in literature. Many remote and underserved areas also have poor telecommunications infrastructure. This leads to obvious challenges with connecting via video or augmented reality. To date, solutions rely on phone conversations and photographs, such as digital postmortem examinations.^1^ True solutions will rely on telecommunication companies to improve service to remote communities.

Limitations of our scoping review include the possibility that we missed literature due to the exclusion criteria or search terms used. Private laboratories may have commercially sensitive information that is not publicly available; hence, we may not have included information on the extent of telepathology used by some companies. In addition, material available on commercial laboratory websites is largely promotional and did not fit the inclusion criteria of our study. For these reasons, company-based writing on the subject may have been excluded, introducing bias. Much information is shared and discussed at national and international meetings and as part of educational webinars, but this information will not have been available for our scoping review. Potential biases of the reviewers, for example, because of personal experiences with telepathology, may also have influenced the way the information was collated and reported.

In conclusion, we found gaps in the literature on the use and validation of telepathology in veterinary medicine despite the widespread use of DM by private veterinary diagnostic laboratories. The literature gap is especially large for its use in gross pathology, and this reflects the limited publication of its use in veterinary medicine. These gaps suggest a need for future research studies focused on validating WSI technology for a wide array of veterinary samples and the utility of real-time telepathology supporting field postmortem examinations. Telepathology has exciting potential, especially in serving remote and underserved communities, but will need refinement and commitment to its development. Clearly, telepathology use is increasing in veterinary medicine, and current and future pathologists must keep pace with the changing technology and identify novel uses to advance the profession.

## Supplemental Material

sj-pdf-1-vdi-10.1177_10406387241241270 – Supplemental material for The use of telepathology in veterinary medicine: a scoping review Supplemental material, sj-pdf-1-vdi-10.1177_10406387241241270 for The use of telepathology in veterinary medicine: a scoping review by Lindsay Rogers, Angelica Galezowski, Heather Ganshorn, Dayna Goldsmith, Carolyn Legge, Katie Waine, Erin Zachar and Jennifer L. Davies in Journal of Veterinary Diagnostic Investigation
